# Associations among plasma markers for N-methyl-d-aspartate receptor hypofunction, redox dysregulation, and insufficient myelination in patients with schizophrenia

**DOI:** 10.1016/j.heliyon.2024.e30193

**Published:** 2024-04-25

**Authors:** Yoshiaki Isomura, Mikiko Ohno, Satoshi Sudo, Mayuko Ono, Yuki Kaminishi, Yukiyoshi Sumi, Atsushi Yoshimura, Kumiko Fujii, Kazufumi Akiyama, Eiichiro Nishi, Yuji Ozeki

**Affiliations:** a Department of Psychiatry, Shiga University of Medical Science, Japan; b Department of Pharmacology, Shiga University of Medical Science, Japan; c Department of Biological Psychiatry and Neuroscience, Dokkyo Medical University School of Medicine, Japan

**Keywords:** Schizophrenia, d-serine, l-serine, Homocysteine, Nardilysin

## Abstract

**Background:**

Several hypotheses regarding the pathomechanisms of schizophrenia have been proposed. If schizophrenia is a unitary disease, then these pathological processes must be linked; however, if such links do not exist, schizophrenia may best be considered a group of disorders. Only a few studies have examined the relationships among these pathomechanisms. Herein, we examined the relationships among deficient myelination, NMDA receptor hypofunction, and metabolic dysregulation by measuring various plasma markers and examining their correlations.

**Methods:**

Plasma samples were collected from 90 patients with schizophrenia and 68 healthy controls. Concentrations of nardilysin (N-arginine dibasic convertase, NRDC), a positive regulator of myelination, the NMDA receptor co-agonist d-serine and glycine, various additional amino acids related to NMDA receptor transmission (glutamate, glutamine, and l-serine), and homocysteine (Hcy), were measured. Concentrations were compared using independent samples *t*-test or logistic regression, and associations were evaluated using Pearson's correlation coefficients.

**Results:**

Plasma glycine (t = 2.05, p = 0.042), l-serine (t = 2.25, p = 0.027), and homocysteine (t = 3.71, p < 0.001) concentrations were significantly higher in patients with schizophrenia compared to those in healthy controls. Logistic regression models using age, sex, smoking status, glutamine, glutamate, glycine, l-serine, d-serine, homocysteine, and NRDC as independent variables revealed significantly lower plasma d-serine (p = 0.024) and NRDC (p = 0.028), but significantly higher l-serine (p = 0.024) and homocysteine (p = 0.001) in patients with schizophrenia. Several unique correlations were found between NMDA receptor-related amino acids and NRDC in patients with schizophrenia compared to those in healthy controls, while no correlations were found between plasma homocysteine and other markers. No associations were found between plasma marker concentrations and disease status or cognitive function in patients with schizophrenia, except for a significant correlation between plasma glycine and full intelligence quotient.

**Conclusion:**

Reduced myelination and NMDA receptor hypofunction may be related to pathological mechanisms in schizophrenia, while homocysteine dysregulation appears to be an independent pathological process. These results suggest that schizophrenia may be a group of disorders with unique or partially overlapping etiologies.

## Highlights

• The concentration of plasma nardilysin (NRDC), a marker for myelination, was reduced in patients with schizophrenia.
• Several plasma markers of NMDA receptor activity were also lower in schizophrenia.
• Plasma concentrations of some markers were uniquely correlated in patients with schizophrenia.
• Plasma homocysteine level was elevated in patients with schizophrenia, but unassociated with other markers.
• Reduced myelination and NMDA receptor hypofunction may be related to schizophrenia pathomechanisms.

## 1 Introduction

According to the Diagnostic and Statistical Manual of Mental Disorders, Fifth Edition Text Revised (DSM-5-TR, American Psychiatric Association 2022), approximately 0.3–0.7 % of the global population is afflicted by schizophrenia [1]. The common symptoms of schizophrenia, including delusions, negative symptoms, and cognitive deficits, interfere with education, social functioning, and employment, resulting in long-term disability. Furthermore, approximately one-third of patients do not respond to available antipsychotic treatments, leading to the prescription of clozapine, an atypical antipsychotic, for treatment-resistant schizophrenia [2]. However, only 40 % of eligible cases respond to clozapine [3]. One reason for these inadequate treatment outcomes is the uncertainty of precise pathogenesis of schizophrenia. Furthermore, while several etiologic hypotheses have been proposed, whether the underlying pathomechanisms are associated or independent remains unclear.

Hypofunction of NMDA-type glutamate receptors (NMDARs) is one of the compelling hypotheses for the pathogenesis of schizophrenia, with supporting evidence accrued over the past two decades. For example, phencyclidine (PCP) at concentrations sufficient to block NMDARs induces neurocognitive and psychotic symptoms in healthy individuals, resembling those of schizophrenia [4]. Moreover, other treatments inducing NMDA receptor blockade are consistently reported to evoke schizophrenia-like symptoms in healthy individuals, or to exacerbate symptoms in patients with schizophrenia [5,6]. In addition, this NMDA receptor hypofunction has been reported to increase dopamine level in the striatum [7,8]. Plasma concentrations of d-serine, an NMDA receptor co-agonist, are reduced in patients with schizophrenia [9]. In addition, abnormalities have also been reported in the NMDA receptor co-agonist glycine and in the d-serine precursor l-serine [10,11].

It is well known that blood homocysteine concentration is elevated in patients with schizophrenia [12]. Homocysteine is a component of one-carbon metabolism, which is involved in synthesizing methyl donors and the antioxidant glutathione (GSH). Thus, abnormalities in homocysteine may disturb normal methylation reactions, including DNA methylation, detoxification of xenobiotics via GSH conjugation, and redox regulation by GSH antioxidant activity [13]. In addition to the dysregulation of these signaling factors, cerebral volume is consistently reported to be lower in patients with schizophrenia [14,15], partly due to a reduction in myelin volume [16]. Nardilysin (N-arginine dibasic convertase, NRDC) is a metalloendopeptidase that positively regulates myelin formation. NRDC-deficient mice exhibited marked thinning of the cerebral cortex and myelin sheaths. Furthermore, the density of NRDC immunoreactive neurons was nearly 50 % higher in the bilateral prefrontal areas of postmortem patients with schizophrenia [17]. However, whether NRDC expression is altered in the living brain of patients with schizophrenia remains unclear.

To address the aforementioned knowledge gaps, this study investigated the relationships among these hypotheses by determining correlations between various plasma biomarker concentrations, including amino acids related to NMDA receptor activity (d-serine, glutamine, glutamic acid, glycine, and l-serine), homocysteine, and NRDC.

## 2 Methods

### 2.1 Participants

Ninety patients with schizophrenia were treated at Dokkyo Medical University Hospital, Mori Hospital, or Shimotsuga General Hospital, and sixty-eight healthy matched controls were recruited between 2010 and 2018. Table 1 summarizes the demographic characteristics of the participants. All patients received antipsychotic medications at an average (± standard deviation) equivalent daily chlorpromazine dose of 765.6 ± 542.3 mg. Two independent psychiatrists evaluated all participants using DSM-IV-TR or DSM-5 criteria. Healthy controls and their first-degree relatives were confirmed to be free of mental illness.

**Table 1:** charactaristics of patients with schizophrenia and healthy controls.

	SZ (n = 90)			HC (n = 68)		
age (years) mean ± SD	48.1	±	12.5	48.5	±	12.1
male/female	47/43			35/33		
smoker	26			13		
duration of illness (years) mean ± SD	24.0	±	12.7			
LTE (years) mean ± SD	11.9	±	2.2			
Antipsychoticsdose(mg/day) mean ± SD	765.6	±	542.3			
(chlorpromazine equivalentdose)						
glutamin (nmol/mL) mean ± SD	481.0	±	60.1	463.0	±	66.7
glutamate (nmol/mL) mean ± SD	55.7	±	27.4	58.3	±	23.2
glycine (nmol/mL)^a^ mean ± SD	212.9	±	50.0	194.9	±	58.4
l-serine (nmol/mL)^a^ mean ± SD	108.2	±	15.6	101.0	±	22.8
d-serine (nmol/mL) mean ± SD	1.50	±	0.50	1.66	±	0.56
homocysteine (nmol/mL)^b^ mean ± SD	14.9	±	9.3	11.0	±	3.2
NRDC (ng/mL) mean ± SD	3.11	±	3.35	3.65	±	3.45
FIQ	92.2	±	10.1			
PANSS	76.9	±	16.7			
BACS-J conposite score (Z-score)	−3.57	±	1.52			

SZ: Schizophrenia, HC: Healthy Controls, LTE: Length of time in education, FIQ: Full scale of Intelligent Quotient, PANSS: Positive And Negative Syndrome Scale, BACS-J: The brief assessment of cognition in schizophrenia Japanese version, SD: Standard Deviation.

a p < 0.05.

b p < 0.01.

### Ethical statement

All participants provided written informed consent after fully explaining the study goals and procedures. The Shiga University of Medical Science Ethics Review Committee approved this study, approval number R2019-306, on July 07, 2021.

### 2.2 Plasma amino acid measurements

An 8-mL sample of whole blood was collected from each participant before noon and placed into BD Vacutainer® CPT™ cell preparation tubes containing sodium heparin (Becton, Dickinson Co., Plainfield, NJ, USA). Plasma samples were isolated from the whole blood samples according to the tube manufacturer's instructions and subsequently stored at −80 °C. The concentrations of glutamine, glutamate, and glycine were measured using an H-class high-performance liquid chromatography (HPLC) system (Waters Corporation) equipped with a quaternary pump, autosampler with the cooler set at <10 °C, column oven, and fluorescence detector. The analytical HPLC column used was Inertsil ODS-4 (2 μm, 2.1 × 100 mm, G.L. Sciences Inc.) with a guard column packed with Inertsil ODS-4 material (2 μm, 2.1 × 10 mm, G.L. Sciences Inc.), maintained at 40 °C. The plasma levels of l-serine and d-serine were also measured using the same HPLC system, except the analytical HPLC column used was Sumichiral OA2500S (5 μm, 4.6 × 250 mm, Sumika Analytical Center), maintained at 40 °C. Amino acids were labeled with 4-fluoro-7-nitro-benzoxazole, while homocysteine was labeled with 4-fluoro-7-sulfamoylbenzofurazan; they were measured according to the protocol presented in a previous study [18]. All chemicals for HPLC analyses were purchased from Sigma-Aldrich Japan (Tokyo, Japan), unless otherwise indicated.

### 2.3 Measurement of serum NRDC

Plasma NRDC concentrations were quantified using a chemiluminescent enzyme immunoassay, as previously described [19], and an automated analyzer (Accuraseed, FUJIFILM Wako Pure Chemical Corporation).

### 2.4 Neuropsychiatric assessments

#### 2.4.1 Brief assessment of cognition in schizophrenia (BACS)-Japanese version

All patients with schizophrenia were evaluated using Version A of the BACS (Japanese version) [20]. This test includes a brief assessment of verbal memory (list learning), a digit sequencing task, a token motor task, a verbal fluency test (category instances and controlled oral word association test), symbol coding, and a Tower of London task. Each of the six measure outcomes were compared to those of healthy controls to generate z-scores. A composite score was then calculated by summing these z-scores and obtaining the z-score of that sum. Healthy control data were obtained from a subgroup of healthy participants (n = 61, 35 males, age = 48.2 ± 11.9).

#### 2.4.2 Premorbid intelligence quotient (I.Q.) assessment

All participants were assessed using the Japanese Adult Reading Test [21,22] to obtain a surrogate premorbid I.Q. estimate.

#### 2.4.3 Positive and negative syndrome scale (PANSS)

The PANSS, a 30-item scale designed by Kay et al. [23], was applied to assess patient symptoms. Performance was scored according to the associated rating manual. Positive (items P1–P7) and negative symptoms (items N1–N7), and general psychopathology (items G1–G16) were assessed by summing the individual item scores for each category.

### 2.5 Statistics

Independent variables of patients and controls were compared using Student's t-test or logistic regression analysis to control for covariates. Associations among variables were evaluated using Pearson's correlation coefficient. In total, 90 correlations were tested; therefore, the significance level was corrected by a factor of 90 using the Bonferroni method. All data were analyzed using SPSS Statistics 25 (SPSS Japan, Inc., Tokyo, Japan). Statistical significance was set at p < 0.05.

## 3 Results

### 3.1 Differences in plasma amino acid concentrations between patients with schizophrenia and healthy controls

Patients with schizophrenia exhibited significantly higher plasma concentrations of glycine (t = 2.05, p = 0.042), l-serine (t = 2.25, p = 0.027) and homocysteine (t = 3.71, p < 0.001) compared to healthy controls (Table 1). Moreover, logistic regression analyses including age, sex, smoking status, glutamine, glutamate, glycine, l-serine, d-serine, homocysteine, and NRDC as independent variables revealed significantly lower plasma concentrations of d-serine (partial regression coefficient ± standard deviation: PRC ± SD = 1.875 ± 0.504, p = 0.024) and NRDC (PRC ± SD = 0.143 ± 0.063, p = 0.028), but significantly higher l-serine (PRC ± SD = −0.028 ± 0.013, p = 0.024) and homocysteine (PRC ± SD = −0.195, p = 0.001) levels in patients with schizophrenia compared to those in healthy controls. No multicollinearity was observed for any of these factors (Table 2).

**Table 2:** Result of logistic regression analysis: effects of each factor in SZ and HC.

	partial regression coefficient (95%CI)		odds ratio(95%CI)		VIF
age	−0.028	(0.053 to −0.061)	0.97	(0.94–1.00)	1.228
sex	−0.496	(0.32 to −1.31)	0.61	(0.27–1.37)	1.228
smoking	−0.393	(0.51 to −1.30)	0.68	(0.27–1.67)	1.119
glutamin (nmol/mL)	−0.004	(0.004 to −0.012)	1.00	(0.99–1.00)	1.138
glutamate (nmol/mL)	0.002	(0.016 to −0.018)	1.00	(0.99–1.00)	1.322
glycine (nmol/mL)	−0.004	(0.004 to −0.012)	0.99	(0.99–1.01)	1.566
l-serine (nmol/mL)^a^	−0.028	(-0.003 to −0.053)	0.97	(0.95–0.99)	1.686
d-serine (nmol/mL)^b^	1.875	(2.86–0.87)	6.52	(2.43–17.49)	1.279
homocysteine (nmol/mL)^b^	−0.195	(-0.078 to −0.312)	0.82	(0.73–0.93)	1.154
NRDC (ng/mL)^a^	0.143	(0.266–0.020)	1.15	(1.02–1.31)	1.150

SZ: Schizophrenia, HC: Healthy Controls, dependent variable: diagnpsis (patient:1, control:2) NRDC: nardilysin; CI: Confidence Interval, VIF: Variance Inflation Factor.

Hosmer-Lemeshow Goodness-of-Fit Test x2 = 4.43 df = 8 p = 0.82.

a p < 0.05.

b p < 0.01.

### 3.2 Correlations between plasma concentrations and demographic factors

Table 3 summarizes the correlations between each measured factor pair. As the correlation coefficients were tested 90 times, the significance level obtained was multiplied by 90 for evaluation. After Bonferroni correction, statistically significant relationships were found between plasma homocysteine concentration and sex (r = −0.481, adjusted p = 0.00297), glutamine and glycine concentrations (r = −0. 469, adjusted p = 0.00495), glutamine and l-serine concentrations (r = 0.571, adjusted p < 0.001), glycine and l-serine concentrations (r = 0.645, adjusted p < 0.001), and l-serine and NRDC concentrations (r = 0.424, adjusted p = 0. 028) within the healthy control group. Among patients with schizophrenia, however, correlations were found only between d-serine concentration and age (r = 0.422, adjusted p = 0.00306), as well as glutamate and d-serine levels (r = −0.375, adjusted p = 0.024).

**Table 3:**
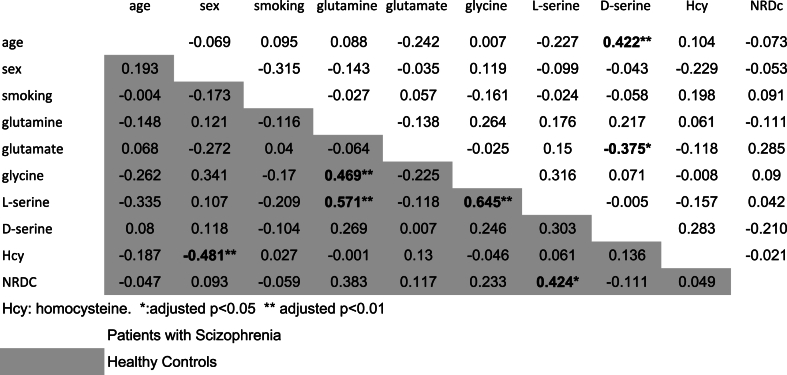
Pearson's correlation coefficient between measured substances.

### 3.3 Correlations between plasma concentrations and symptom measures

The psychiatric symptoms, full intelligence quotient (FIQ), and cognitive function in patients with schizophrenia were assessed using the PANSS, the Japanese Adult Reading Test (JART), and the BACS-J, respectively. Each of these measures was used as the dependent variable in multiple regression models with age, sex, smoking status, duration of illness, educational history, chlorpromazine equivalents of antipsychotic medications, glutamine, glutamic acid, glycine, l-serine, d-serine, homocysteine, and NRDC as independent variables. Both FIQ and BACS-J scores were significantly correlated with the duration of illness and education. Furthermore, FIQ was correlated with plasma glycine concentration, while no significant correlations were found between JART and any other aforementioned factors (Table 4).

**Table 4:** Relationship between the patient's condition and each factor: Multiple regression analysis.

FIQ: Full Intelligent Quotient				
	partial regression coefficient (95%CI)		p-value	VIF
age	0.26	(−0.003–0.51)	0.05	3.29
male/female	−3.47	(−7.50–0.56)	0.09	1.30
smoker	−2.39	(−6.70–1.91)	0.27	1.22
duration of illness (years)	−0.34	(−0.59 to −0.10)	0.01	3.08
LTE (years)	2.32	(1.4–3.2)	<0.01	1.22
Antipsychoticsdose (mg/day)	<0.01	(−0.0030–0.004)	0.83	1.29
glutamin (nmol/mL)	<0.01	(−0.03–0.03)	0.94	1.20
glutamate (nmol/mL)	0.01	(−0.06–0.09)	0.73	1.34
glycine (nmol/mL)	0.05	(0.006–0.09)	0.02	1.28
l-serine (nmol/mL)	0.00	(−0.14–0.13)	0.99	1.40
d-serine (nmol/mL)	−0.38	(−4.8–4.05)	0.87	1.58
homocysteine (nmol/mL)	−0.08	(−0.30–0.14)	0.48	1.31
NRDC (ng/mL)	−0.17	(−0.76–0.41)	0.56	1.21

LTE: Length of time in education, antipsychotics dose means chlorpromazine equivalent dose VIF: Variance Inflation Factor, NRDC: nardilysin adjusted R^2^ = 0.311 F value = 4.08 p < 0.01.				

PANSS: Positive and Negative Syndrome Scale				
	partial regression coefficient (95%CI)		p-value	VIF
age	−0.30	(−0.78–0.19)	0.23	3.29
male/female	−5.56	(−13.1–2.01)	0.15	1.30
smoker	−6.27	(−14.4–1.8)	0.13	1.22
duration of illness (years)	0.23	(−0.23–0.69)	0.33	3.08
LTE (years)	−1.38	(−3.1–0.3)	0.11	1.22
Antipsychoticsdose (mg/day)	0.01	(−0.002–0.012)	0.18	1.29
glutamin (nmol/mL)	−0.03	(−0.086–0.035)	0.40	1.20
glutamate (nmol/mL)	−0.08	(−0.22–0.062)	0.27	1.34
glycine (nmol/mL)	0.02	(−0.056–0.095)	0.61	1.28
l-serine (nmol/mL)	0.17	(−0.080–0.426)	0.18	1.40
d-serine (nmol/mL)	−4.60	(−12.92–3.72)	0.27	1.58
homocysteine (nmol/mL)	0.27	(−0.14–0.69)	0.19	1.31
NRDC (ng/mL)	−0.17	(−1.27–0.92)	0.75	1.21

LTE: Length of time in education, antipsychotics dose means chlorpromazine equivalent dose VIF: Variance Inflation Factor, NRDC: nardilysin adjusted R2 = 0.101 F value = 1.769 p = 0.064.				

BACS-J: Brief Assessment of Cognitive Scale-Japanese version				
	partial regression coefficient (95%CI)		p-value	VIF
age	0.016	(−0.03–0.06)	0.54	3.29
male/female	−0.511	(−1.19–0.17)	0.61	1.30
smoker	0.619	(−0.11–1.35)	0.14	1.22
duration of illness (years)	−0.042	(−0.08 to −0.00)	0.01	3.08
LTE (years)	0.176	(0.03–0.33)	0.03	1.22
Antipsychoticsdose (mg/day)	0.000	(−0.001–0.000)	0.89	1.29
glutamin (nmol/mL)	−0.001	(−0.006–0.005)	0.89	1.20
glutamate (nmol/mL)	0.004	(−0.008–0.017)	0.70	1.34
glycine (nmol/mL)	0.004	(−0.003–0.011)	0.29	1.28
l-serine (nmol/mL)	−0.013	(−0.035–0.010)	0.19	1.40
d-serine (nmol/mL)	−0.041	(−0.79–0.71)	0.64	1.58
homocysteine (nmol/mL)	−0.010	(−0.05–0.03)	0.74	1.31
NRDC (ng/mL)	0.026	(−0.07–0.13)	0.46	1.21

LTE: Length of time in education, antipsychotics dose means chlorpromazine equivalent dose VIF: Variance Inflation Factor, NRDC: nardilysin adjusted R^2^ = 0.25 F value = 1.97 p = 0.035.

## 4 Discussion

Several hypotheses regarding the pathomechanisms of schizophrenia have been proposed. The present study aimed to determine the associations between three major etiological hypotheses of schizophrenia (reduced myelination, NMDA receptor hypofunction, and metabolic disruption/redox dysregulation), by measuring factors suggestive or indicative of each related pathomechanism. As in previous studies, homocysteine level was found to be higher in patients with schizophrenia than in healthy controls, as revealed by univariate analysis, suggesting disruption of normal one-carbon metabolism and ensuing dysregulation of DNA methylation and GSH function (detoxification and antioxidant activity). Similarly, we found significant differences in plasma concentrations of glycine and l-serine, as assessed by univariate analysis (t-tests). In contrast to previous studies, no difference in plasma d-serine level was found between patients with schizophrenia and controls, as assessed by univariate analysis. However, logistic regression analysis revealed higher levels of l-serine and homocysteine, but lower levels of d-serine and NRDC in patients with schizophrenia compared to those in controls. Many correlations between various factors found in control participants (between homocysteine and sex, glutamine, and glycine; glutamine and l-serine; glycine and l-serine; and NRDC and l-serine) were not found in patients with schizophrenia, although significant correlations between d-serine level and age, as well as between d-serine and glutamate levels, were found. However, none of these factors were correlated with disease status or cognitive function (PANSS, BACS-J, and JART results), except for a significant correlation between plasma glycine concentration and FIQ. Collectively, these findings suggest that NMDA receptor hypofunction, as evidenced by low d-serine level, and myelination deficiency, as evidenced by low NRDC level, are linked in patients with schizophrenia, while abnormalities in brain metabolism and redox regulation, as evidenced by elevated homocysteine level, may be independent pathomechanisms. Thus, these results indicate that schizophrenia may be a group of disorders with unique or partially overlapping etiologies.

Many studies have found higher plasma homocysteine level in patients with schizophrenia than in healthy controls [12,24]. Homocysteine is a component of one-carbon metabolism, contributing a methyl group for methionine synthesis. Ultimately, this process leads to the formation of S-adenosylmethionine, which acts as a methyl group donor in a variety of biological processes, including regulation of gene expression through DNA methylation. Indeed, abnormalities in DNA methylation have been reported in schizophrenia [25], indicating that homocysteine elevation may be linked to disease pathogenesis via dysregulation of gene expression networks [26]. Proton magnetic resonance spectroscopy (HMRS) has also revealed reduced glutathione level in the brain parenchyma of patients with schizophrenia [27], as well as lower plasma/serum glutathione levels [28]. Glutathione, synthesized from homocysteine [29], is among the most important antioxidants in brain tissue. Furthermore, in the liver, the homocysteine-dependent *trans*-sulfation pathway is important for maintaining the intracellular glutathione pool, and regulation of this pathway has been shown to be critical under oxidative stress conditions. Further, research has shown that abnormalities in this pathway may impair the redox buffering capacity of the cell [30]. A quantitatively important relationship has been shown between homocysteine metabolism and glutathione synthesis in the *trans*-sulfation pathway and its regulation by redox changes [30]. Therefore, these findings suggest that the pathology of schizophrenia may be associated with alterations in glutathione synthesis and brain redox regulation. However, these pathways appear to be unrelated to myelination deficits and NMDA receptor hypofunction.

Prior research has indicated that d-serine may contribute to the pathogenesis of schizophrenia due to its role as an NMDA receptor co-agonist [4,5]. Substances that decrease the function of NMDA receptors cause both negative and positive symptoms of schizophrenia. Since d-serine is a co-agonist of NMDA receptors, it has long been suggested that a decrease in blood levels of d-serine may cause a decrease in NMDA receptor function, thereby triggering the development of schizophrenia symptoms [[4], [5], [6],^31^,^32^]. A recent meta-analysis concluded that d-serine level is significantly reduced in patients with schizophrenia [9]. The current results are thus partially consistent with the d-serine insufficiency hypothesis. Abnormality in l-serine, which is converted to d-serine by the schizophrenia-associated gene serine racemase, has also been reported, but results across studies are less consistent [11]. Moreover, plasma l-serine level was found to be correlated with plasma NRDC level in healthy controls, suggesting a link between NMDA receptor hypofunction and myelination deficits in the pathogenesis of schizophrenia.

Nardilysin is an M16 family metalloendopeptidase implicated in myelination, cancer growth, and inflammation [[33], [34], [35]]. Schizophrenia is linked to neurodevelopmental abnormalities, including dysregulation of myelination [36]. Oligodendrocyte density was decreased in a region-specific manner in patients with bipolar disorder and schizophrenia [37]. Expression of the myelin oligodendrocyte glycoprotein (MOG) gene, a target antigen for myelin-destructive antibodies in autoimmune CNS demyelinating diseases, such as multiple sclerosis, and a cell surface marker of oligodendrocyte maturation, was also significantly decreased in schizophrenia [38]. Thus, demyelination may occur in patients with schizophrenia. The low levels of plasma NRDC observed among patients with schizophrenia may be related to these neurodevelopmental abnormalities. Furthermore, NRG1 is involved in the mechanisms by which NRDC controls myelin sheath formation, and one study proposed that NRG1 may be involved in the pathogenesis of schizophrenia [39]. Following the results of this study, a genome-wide association study failed to identify a link between NRG1 and schizophrenia, although it is believed that this does not entirely rule out a potential link [40]. In contrast, the low level of NRDC observed in our study infers an interesting relationship between NRG1 and schizophrenia.

This study measured substances associated with altered DNA methylation, NMDA receptor hypofunction, and insufficient myelination, all of which have been linked to the pathophysiology of schizophrenia. Plasma NRDC and l-serine concentrations were found to be significantly correlated among healthy controls, but not in patients with schizophrenia, suggesting that some relationships between myelin formation and NMDA receptor function may be disrupted in schizophrenia. Similarly, differences in the plasma amino acid correlation pattern between healthy controls and patients with schizophrenia suggest that metabolic pathways, including those necessary for NMDA receptor function, are impaired in patients with schizophrenia. In contrast, no correlations were found between plasma homocysteine and the plasma concentrations of multiple NMDA receptor-associated amino acids, indicating that homocysteine-related pathomechanisms, such as aberrant DNA methylation and glutathione metabolism (as well as the ensuing abnormalities in gene expression and/or redox regulation), may independently contribute to schizophrenia development.

Overall, we found no significant relationships between cognitive function or clinical symptoms and the plasma substances measured in this study, with the exception of that between FIQ and glycine. Further, some reports have indicated an association between plasma/serum glycine levels and schizophrenia, but the results are inconsistent [11]. As such, further investigations are required to confirm the relationship between plasma glycine levels and neurocognitive functions in schizophrenia.

## 5 Limitations

Several limitations should be considered when interpreting these findings. First, the cross-sectional study design limits speculation on causality. Second, the schizophrenia group comprised patients with variable disease duration and prior exposure to different antipsychotics, factors that may influence plasma concentrations of the measured substances. The group is heterogeneous, including patients with stable states. To overcome these limitations, further longitudinal studies in patients with first-episode psychosis who are not taking medication are needed. Third, all substances were measured in the plasma, and it is unclear whether these measurements accurately reflect central nervous system concentrations or bioavailability. Fourth, regarding oxidative stress markers, 2-thiobarbituric acid reactive substances (TBARS), asymmetric dimethylarginine (ADMA), and total antioxidation capacity (TAC) were not measured in this study. Finally, numerous additional substances potentially indicative of these and other pathomechanisms were not examined.

## 6 Conclusions

Several major pathomechanisms have been proposed to explain schizophrenia onset, progression, symptoms, and treatment response. While there are several widely accepted core features of this disease, schizophrenia remains a highly heterogeneous disorder, suggesting that these pathomechanisms may evolve independently. Therefore, schizophrenia may be considered a cluster of disorders, rather than a unitary disorder. Alternatively, it is possible that distinct pathogenic processes may lead to a common disorder. Our correlation analyses suggest a link between neurodevelopmental abnormalities, as evidenced by reduced plasma concentrations of the myelination-promoting protein NRDC and NMDA receptor hypofunction as evidenced by reduced plasma d-serine. In contrast, our results indicate that homocysteine dysregulation may occur independent of these other pathomechanisms. The present results are not an exhaustive review of pathophysiologically relevant substances; however, the limited measurements predicted that the pathophysiology of schizophrenia may be multifaceted.

Further research on the relationship between markers of different pathological processes implicated in schizophrenia could help to clarify the common and unique etiologies of schizophrenia in individual patients to ensure more effective targeted therapy.

## Ethics statement

We understand and comply with the Duties of Authors set forth in the Elsevier policies (https://www.elsevier.com/about/policies-and-standards/publishing-ethics).

## Financial

The authors declare no known competing financial interests or personal relationships that could potentially influence the work reported in this paper.

## Data availability statement

The data supporting this study's findings are available on request from the corresponding author, [Y.O.]. The data are not publicly available due to their containing information that could compromise the privacy of research participants. Furthermore, for the use of data, the regulation requires the permission of this institution's Ethics Committee.

## CRediT authorship contribution statement

**Yoshiaki Isomura:** Writing – original draft, Data curation. **Mikiko Ohno:** Methodology, Investigation. **Satoshi Sudo:** Validation. **Mayuko Ono:** Validation. **Yuki Kaminishi:** Validation. **Yukiyoshi Sumi:** Validation. **Atsushi Yoshimura:** Validation. **Kumiko Fujii:** Validation, Methodology, Data curation. **Kazufumi Akiyama:** Validation, Data curation. **Eiichiro Nishi:** Methodology, Investigation. **Yuji Ozeki:** Writing – review & editing, Writing – original draft, Supervision, Funding acquisition, Data curation, Conceptualization.

## Declaration of competing interest

The authors declare that they have no known competing financial interests or personal relationships that could have appeared to influence the work reported in this paper.
